# Dual Effect of Beta-Amyloid on α7 and α4β2 Nicotinic Receptors Controlling the Release of Glutamate, Aspartate and GABA in Rat Hippocampus

**DOI:** 10.1371/journal.pone.0029661

**Published:** 2012-01-11

**Authors:** Elisa Mura, Stefania Zappettini, Stefania Preda, Fabrizio Biundo, Cristina Lanni, Massimo Grilli, Anna Cavallero, Guendalina Olivero, Alessia Salamone, Stefano Govoni, Mario Marchi

**Affiliations:** 1 Department of Drug Sciences, Centre of Excellence in Applied Biology, University of Pavia, Pavia, Italy; 2 Section of Pharmacology and Toxicology, Department of Experimental Medicine, University of Genoa, Genoa, Italy; 3 Centre of Excellence for Biomedical Research, University of Genoa, Genoa, Italy; Biological Research Center of the Hungarian Academy of Sciences, Hungary

## Abstract

**Background:**

We previously showed that beta-amyloid (Aβ), a peptide considered as relevant to Alzheimer's Disease, is able to act as a neuromodulator affecting neurotransmitter release in absence of evident sign of neurotoxicity in two different rat brain areas. In this paper we focused on the hippocampus, a brain area which is sensitive to Alzheimer's Disease pathology, evaluating the effect of Aβ (at different concentrations) on the neurotransmitter release stimulated by the activation of pre-synaptic cholinergic nicotinic receptors (nAChRs, α4β2 and α7 subtypes). Particularly, we focused on some neurotransmitters that are usually involved in learning and memory: glutamate, aspartate and GABA.

**Methodology/Findings:**

We used a dual approach: *in vivo* experiments (microdialysis technique on freely moving rats) in parallel to *in vitro* experiments (isolated nerve endings derived from rat hippocampus). Both *in vivo* and *in vitro* the administration of nicotine stimulated an overflow of aspartate, glutamate and GABA. This effect was greatly inhibited by the highest concentrations of Aβ considered (10 µM *in vivo* and 100 nM *in vitro*). *In vivo* administration of 100 nM Aβ (the lowest concentration considered) potentiated the GABA overflow evoked by nicotine. All these effects were specific for Aβ and for nicotinic secretory stimuli. The *in vitro* administration of either choline or 5-Iodo-A-85380 dihydrochloride (α7 and α4β2 nAChRs selective agonists, respectively) elicited the hippocampal release of aspartate, glutamate, and GABA. High Aβ concentrations (100 nM) inhibited the overflow of all three neurotransmitters evoked by both choline and 5-Iodo-A-85380 dihydrochloride. On the contrary, low Aβ concentrations (1 nM and 100 pM) selectively acted on α7 subtypes potentiating the choline-induced release of both aspartate and glutamate, but not the one of GABA.

**Conclusions/Significance:**

The results reinforce the concept that Aβ has relevant neuromodulatory effects, which may span from facilitation to inhibition of stimulated release depending upon the concentration used.

## Introduction

In 1984 Glenner and Wong sequenced a small peptide isolated from the brains of Alzheimer's disease (AD) patients. This peptide, known as beta-amyloid (Aβ), was subsequently recognized as the main pathogenetic marker of AD [1]. The increased levels of the peptide in extracellular sites lead to subsequent events that include the aggregation and deposition of Aβ, the hyperphosphorylation of tau protein, the occurrence of neurotoxic phenomena and consequent neuronal death and, finally, dementia [2], [3].

Although the neurotoxic role of Aβ is unchallenged, the scientific community has emphasized the existence of physiological roles for the peptide (as reviewed in [4]). The idea is that Aβ may be important in normal brain functioning, but when it exceeds certain concentrations the peptide may become neurotoxic. Both different aggregates and isoforms of Aβ may have different biological actions in a continuum from physiology to pathology determining and participating to the subsequent stages of the disease [4]. In this regard, we previously showed that Aβ acts as a neuromodulator, affecting neurotransmitter release in the absence of evident signs of neurotoxicity [5]–[7]. This role may be at borderline between physiology and pathology. In a physiological context, the neuromodulatory role of Aβ would be important for the proper balance among neurotransmitter systems. On the contrary, in pathological conditions the Aβ-mediated synaptic modulation might be related to Aβ-driven functional alterations of neurotransmission in addition and before the well known neurodegenerative events. The dysregulation of neurotransmission may in turn produce early cognitive and non-cognitive disturbances based on the neurotransmitter systems and the brain area involved.

For all these reasons, we started to systematically explore the effect of the peptide on different brain areas and neurotransmitter systems both *in vivo* and *in vitro*. Our previous studies showed that Aβ inhibits the cholinergic control of both dopamine and γ-aminobutyric acid (GABA) release in the nucleus accumbens and caudate putamen [5]–[7]. In these brain areas, Aβ affects neurotransmitter release acting downstream muscarinic receptors, particularly on protein kinase C [5].

The purpose of the present study is to evaluate whether nicotinic acetylcholine receptors (nAChRs) may also be involved in the neuromodulatory action of Aβ. In this regard, several findings suggest that Aβ interacts with high affinity with nAChRs. The peptide can bind to α7 and with 100–5000 times lower affinity to α4β2 nAChRs [8]. Interestingly, the Aβ-nAChRs interaction may serve non-neurotoxic roles (as the control of synaptic plasticity and neuronal homeostasis), as well as contributing to AD etiology [9]. There are conflicting data concerning the type of effect exerted by Aβ on nAChRs, with some authors showing the activation of the receptor, whereas others report an antagonist action [10]. These differences may be related either to the experimental model investigated or to the Aβ species and concentrations administered [11].

An intriguing brain area for the purpose of this study is the hippocampus, a neuroanatomical structure that is implicated in learning and memory and that is involved in AD from the early stages of the disease [12]. Cholinergic projections to the hippocampus mainly derive from the medial septum-diagonal band via the fimbria fornix [13], [14]. Acetylcholine released from these projections acts on both muscarinic and nicotinic targets modulating neurotransmission [15], [16]. Interestingly, we recently demonstrated that the activation of both α7 and α4β2 nAChRs subtypes promotes the hippocampal release of inhibitory and excitatory neurotransmitters such as GABA, glutamate (Glu), and aspartate (Asp) [17], [18] This cholinergic control of GABAergic and glutamatergic systems may have important effects concerning the intra-hippocampal circuits modulating synaptic plasticity, a process relevant to memory trace formation [19]. Moreover, a putative modulatory effect of Aβ on excitatory and inhibitory transmitters may be relevant in the derangements of synaptic activity preceding and accompanying neurodegenerative processes associated with Aβ deposition in the course of the disease.

We used only Aβ1–40 peptide in our experiments, for two main reasons. First, physiologically the 40-amino-acid long peptide is the most abundant form [20]–[22]. Second, Aβ1–42 has been reported to aggregate faster than Aβ1–40 [23] and thus it is considered as the most neurotoxic species [24]. With the aim of exploring new effects of Aβ other than the neurotoxic ones, we chose to avoid this potentially confounding element.

For all these reasons, using both *in vivo* (microdialysis) and *in vitro* (synaptosomes in superfusion) techniques, we studied whether Aβ1–40 (pM-µM) may affect the nicotine (Nic)-evoked release of GABA, Glu and Asp in hippocampus. In particular, we evaluated whether the neuromodulatory action of Aβ may be exerted on both α7 and α4β2 nAChRs subtypes.

The results presented here show the Aβ capability to regulate the nicotinic control of aspartate, glutamate and GABA release in absence of evident signs of neurotoxicity, and in a way that depends upon the concentration used and the nicotinic receptor subtype involved.

## Results

### *In vivo* results

**Figure 1:**
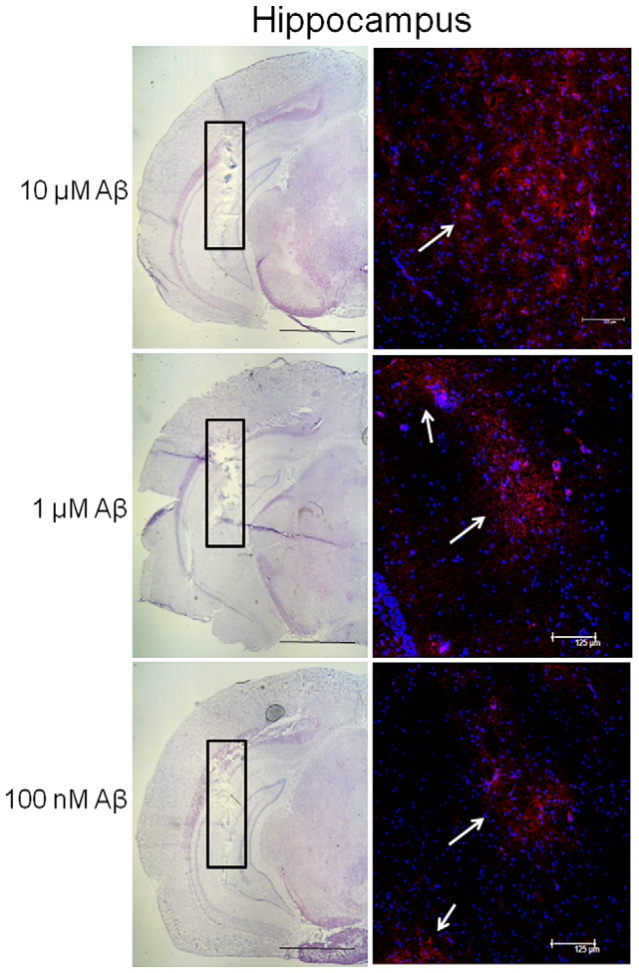
Immunohistochemical analysis showing beta-amyloid (Aβ) content in hippocampal tissue after its administration at different concentrations. Coronal section indicating the location of the probe (hippocampus, black rectangle) counterstained with Mayer Hematoxylin and relative fluorescence micrographs of the area within the black rectangle showing the presence of human Aβ protein. Aβ immunoreactivity (red-PE staining, white arrows) immediately after perfusion of 10 µM, 1 µM and 100 nM Aβ1–40. Nuclear DNA was counterstained with Hoechst 33342 (blue staining). Scale bars for Mayer hematoxylin sections: 200 µm. Scale bars for fluorescent micrographs: 125 µm.

We first performed immunohistochemical analysis in order to test whether the administration of Aβ1–40 through the dialysis probe allowed the delivery of the peptide to the tissue. Fig. 1 shows the presence of the peptide for all the concentrations tested (100 nM–10 µM) within the hippocampus. As expected, there was a visible positive correlation between the concentration administered and the signal of Aβ immunoreactivity in the tissue.

We characterized the Aβ peptide conformation by using Western Blot procedure, starting from the stock solution (100 µM Aβ1–40 solution). With SDS-PAGE, all Aβ1–40 preparations analyzed resolved to immunoreactive species consistent with Aβ monomer (Fig. S1).

We then analyzed the effect of an acute administration of Nic on the release of GABA, Glu, and Asp in hippocampus *in vivo*. Previously published data have shown that the administration by microdialysis of 20 mM Nic is not enough to evoke a GABA overflow in hippocampus [25]. In similar experimental conditions Toth [26] showed that 50 mM Nic is able to significantly increase the levels of Glu and Asp in hippocampal extracellular compartment. Despite Toth [26] was able to obtain a substantial release using lower concentrations, in our *in vivo* experiments the preliminary concentration curve performed (not shown) indicated 50 mM Nic as the best working condition. Hence, we evaluated 50 mM Nic. It should be stressed that microdialysis application of drugs can be considered as a point source in the brain, having a sphere of action with decreasing drug concentration. The effective concentrations used in the *in vivo* experiments cannot be specified; hence the concentration in the probe was given. Therefore the current drug concentration could be supraphysiological [27]. Future experiments comparing other administration methods may help to provide insight on this aspect.

**Figure 2:**
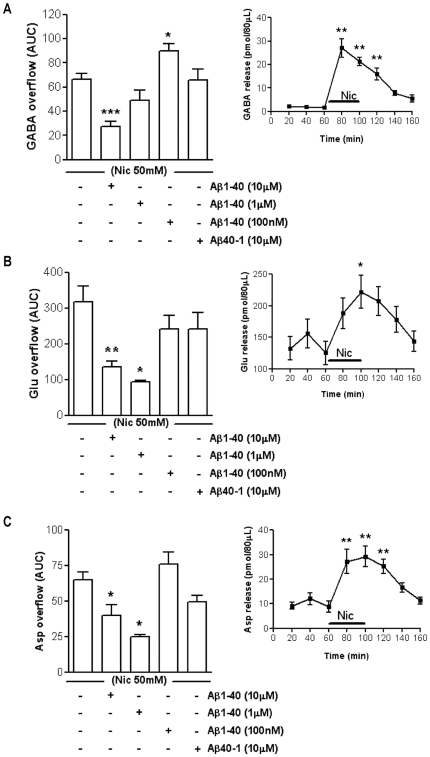
*In vivo* effect of different concentrations of beta-amyloid on the nicotine-induced overflow of hippocampal neurotransmitters. Effect of beta-amyloid (Aβ)1–40 (100 nM–10 µM) on 50 mM nicotine (Nic)-induced overflow of GABA (**A**), glutamate (Glu, **B**) and aspartate (Asp, **C**). *p<0.05, **p<0.01, ***p<0.001 vs. Nic (One-way ANOVA followed by Dunnett's Multiple Comparison Test). Data are expressed as mean ± SEM of 4–15 individual rats for each experimental group. Insets show the time course of 50 mM Nic-induced release of GABA (**A inset**), Glu (**B inset**) and Asp (**C inset**). *p<0.05, **p<0.01 vs. basal release (One-way ANOVA followed by Bonferroni *post hoc* test). Data are expressed as mean ± SEM of 13–15 individual rats.

In our experimental conditions a 40 minutes-long administration of 50 mM Nic was able to greatly enhanced GABA release from basal values. The effect of Nic peaked after 20 minutes of perfusion (1321%, Fig. 2A inset) and persisted for additional 20 minutes after the end of the treatment (+742% compared to basal values, Fig. 2A inset). Moreover, a 40 minutes-long treatment with 50 mM Nic was also effective in stimulating the release of excitatory amino acids, thus supporting the previously published data. Concerning Glu, the peak effect of Nic was observed at the end of the treatment (61%), then the release returned to the basal level (Fig. 2B inset). The time course of the endogenous Asp release evoked by Nic (50 mM) *in vivo* is reported in the inset to Fig. 2C. The effect of Nic on Asp release reached the top at the end of the perfusion with Nic (190%).

Based on the previously described time courses, we analyzed the effect of Nic exposure on the cumulative amount of neurotransmitter released over time, calculating the area under the curve (AUC). Then, we compared the average AUC after 50 mM Nic exposure to the average AUCs obtained after the separate administration of Nic in co-perfusion with Aβ1–40. Particularly, we created a dose-response curve evaluating different concentrations of the peptide (100 nM–10 µM). The Nic-evoked GABA overflow was inhibited by 10 µM Aβ1–40 (59%) and potentiated by 100 nM Aβ1–40 (35%) (Fig. 2A). Concerning 1 µM Aβ1–40 (the middle concentration evaluated *in vivo*), there was a trend to exert an inhibitory effect which, however, did not reach the statistical significance (Fig. 2A). As far as excitatory neurotransmitters, 10 µM Aβ1–40 was able to inhibit the Nic-induced overflow of both Glu (57%, Fig. 2B) and Asp (38%, Fig. 2C). Also 1 µM Aβ1–40 impaired the Nic-induced overflow of both Glu (70%) and Asp (61%), whereas 100 nM Aβ1–40 was ineffective (Fig. 2B and 2C). The reverse peptide Aβ40–1 (tested at 10 µM) did not modify the Nic-induced release of GABA (Fig. 2A), Glu (Fig. 2B) and Asp (Fig. 2C). None of the concentrations of Aβ1–40 tested *in vivo* (100 nM, 1 µM and 10 µM) affected the basal level of GABA, Glu and Asp in the hippocampus (Fig. S2).

**Figure 3:**
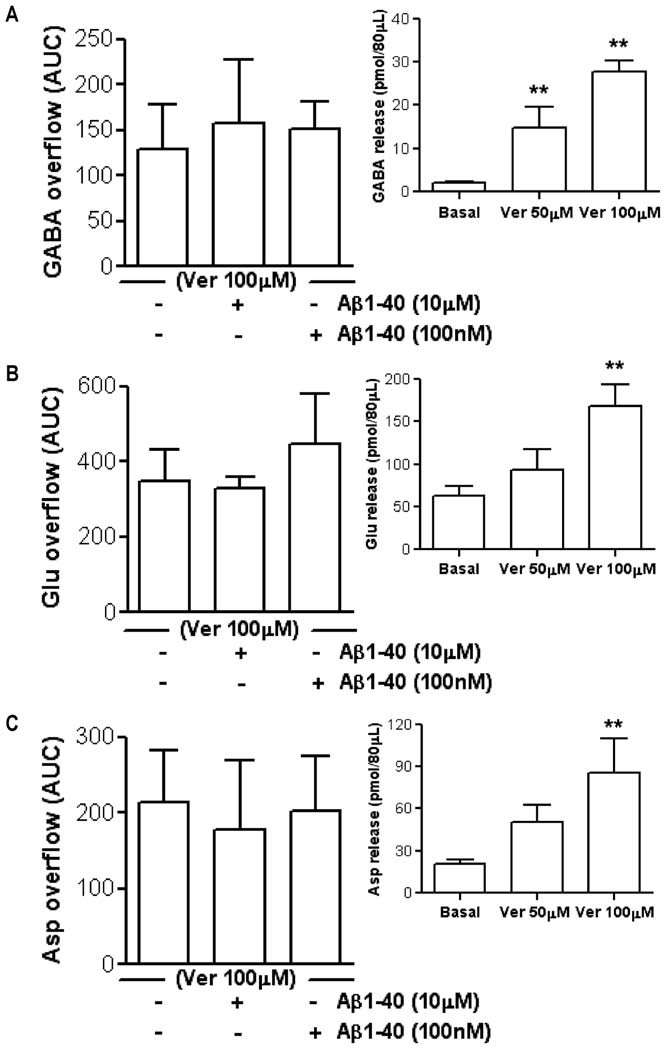
Lack of effect of beta-amyloid on the veratridine-induced neurotransmitter release in hippocampus *in vivo*. Effect of beta-amyloid (Aβ)1–40 (100 nM and 10 µM) on 100 µM veratridine (Ver)-induced overflow of GABA (**A**), glutamate (Glu, **B**) and aspartate (Asp, **C**). (One-way ANOVA followed by Dunnett's Multiple Comparison Test). Data are expressed as mean ± SEM of 4 individual rats for each experimental group. Insets show the effect of two different concentrations of Ver (50 µM and 100 µM) on the release of GABA (**A inset**), Glu (**B inset**) and Asp (**C inset**). **p<0.01 vs. Basal (One-way ANOVA followed by Dunnett's Multiple Comparison Test). Data are expressed as mean ± SEM of 4–9 individual rats for each experimental group.

In order to evaluate whether the effects of Aβ1–40 were selective for the nicotinic control of neurotransmitter release, we chose veratridine (Ver) as another secretory stimulus. Based on previous microdialysis publications [28], [29], we evaluated two different concentrations for this depolarizing stimulus. As shown in Fig. 3A–3C insets, 100 µM Ver elicited the release of the three neurotransmitters studied although to a different extent (GABA = 1253%; Glu = 170%; Asp = 309%). The lower concentration of Ver (50 µM) was able to elicit the release of GABA (615%) but not the one of Glu and Asp. For these reasons we used 100 µM Ver in the following experiments. We then evaluated the effect of Aβ1–40 at 100 nM and 10 µM (the lowest and highest concentration that had been considered *in vivo*, respectively) on the release of neurotransmitters evoked by 100 µM Ver, comparing the specific AUC. Both of these two concentrations of Aβ1–40 did not affect the Ver-induced overflow of GABA, Glu, and Asp (Fig. 3A, Fig. 3B and Fig. 3C, respectively).

### *In vitro* results

**Figure 4:**
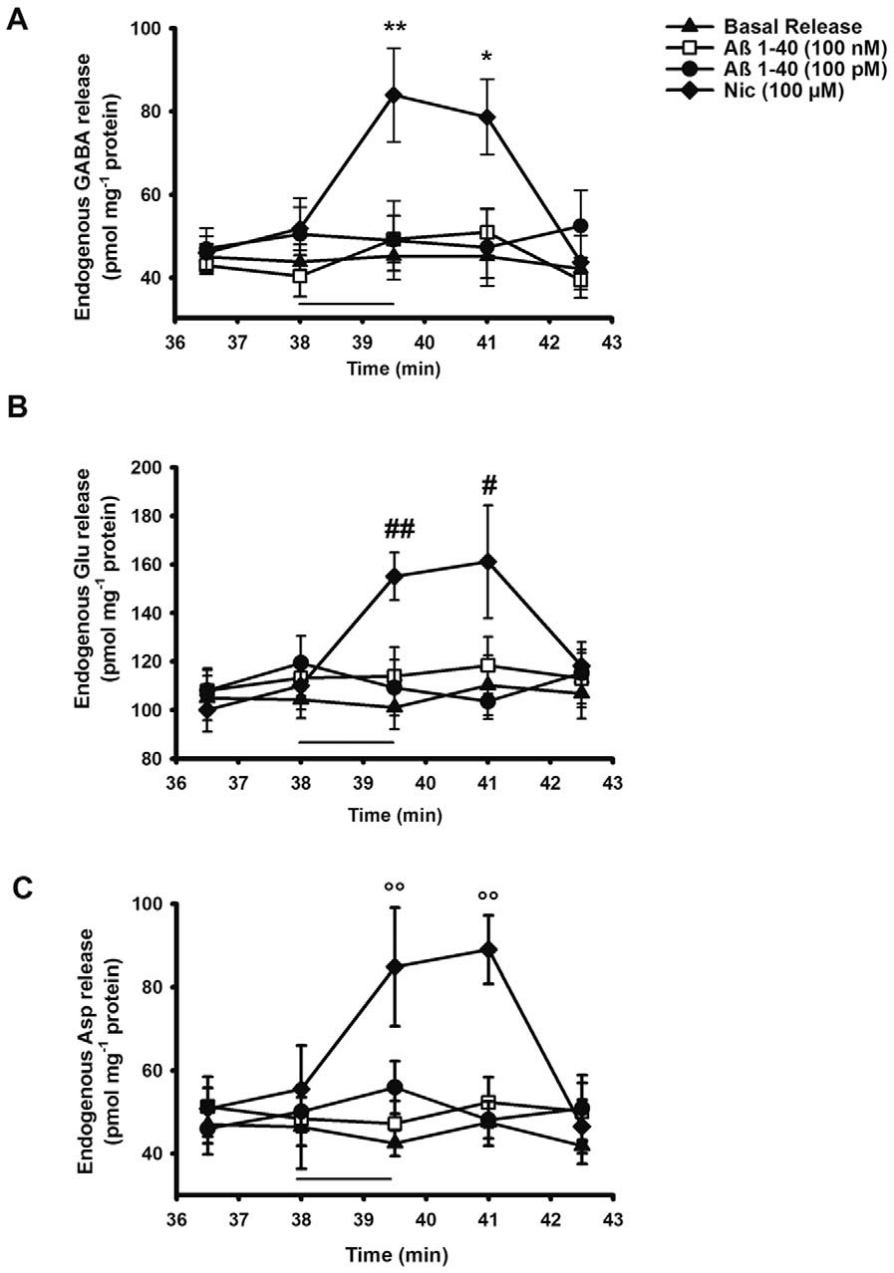
Time course of nicotine and beta-amyloid evoked GABA (A), glutamate (B) and aspartate (C) release *in vitro*. Synaptosomes were depolarized with nicotine (Nic) or beta-amyloid (Aβ)1–40 for 90 s at *t* = 38 min of superfusion (black bar). Values represent mean ± SEM of at least eight replicate superfusion chambers per condition (basal or evoked release). *p<0.05, **p<0.01 vs. GABA basal release; ^#^p<0.05; ^##^p<0.01 vs. glutamate (Glu) basal release; ^°°^p<0.01 vs. aspartate (Asp) basal release. Two way ANOVA followed by Bonferroni *post hoc* test.

Likewise, Nic was able to evoke an overflow of GABA, Glu, and Asp from hippocampal nerve endings *in vitro* experiments. Figures 4A, 4B, and 4C respectively show the time course of the endogenous GABA, Glu, and Asp release enhanced by 100 µM Nic. Concerning GABA release, the peak effect (60%) of the nicotinic agonist was observed at the end of the 90 s-long treatment. In the case of both Asp and Glu the maximal effect of Nic (60% and 50% respectively) was reached in fraction 41 (about 3 minutes after having stimulated the synaptosomes with the cholinergic agonist), after which the release returns to the basal level.

Figures 4A, 4B, and 4C also show that the presence of Aβ1–40 in the medium of perfusion did not modify basal release of all three selected neurotransmitters.

**Figure 5:**
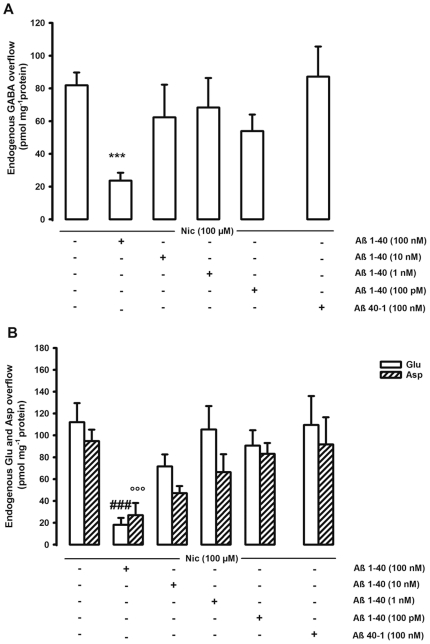
*In vitro* effect of beta-amyloid on the nicotine-induced overflow of hippocampal neurotransmitters. Effect of different concentrations of beta-amyloid (Aβ)1–40 on the nicotine (Nic)-induced overflow of endogenous GABA (**A**), glutamate and aspartate (Glu and Asp respectively, **B**) from rat hippocampal synaptosomes. Synaptosomes were depolarized with Nic for 90 s at *t* = 38 min of superfusion. When appropriate Aβ was introduced 8 min before Nic. Data are mean ± SEM of 5–8 experiments run in triplicate. ***p<0.001 vs. Nic-evoked GABA overflow; ^###^p<0.001 vs. Nic-evoked Glu overflow; ^°°°^p<0.001 vs. Nic-evoked Asp overflow. One way ANOVA followed by Dunnett *post hoc* test.

We then evaluated the effects of different concentrations of Aβ1–40 (from 100 pM up to 100 nM) on the Nic-evoked overflow of GABA, Glu, and Asp. Fig. 5 shows that the highest concentration of Aβ1–40 tested *in vitro* (100 nM) greatly inhibited the Nic-induced overflow of GABA (70%), Glu (85%), and Asp (70%), whereas all the other concentrations (10 nM, 1 nM, 100 pM) were ineffective. Therefore, *in vitro* the Nic-induced GABA overflow was not potentiated by low concentrations of the peptide, as observed *in vivo*. The inhibitory effect was specific for Aβ1–40 since the reverse peptide (Aβ40–1) at the same concentration (100 nM) did not modify the Nic-stimulated release of all three neurotransmitters (Fig. 5).

**Figure 6:**
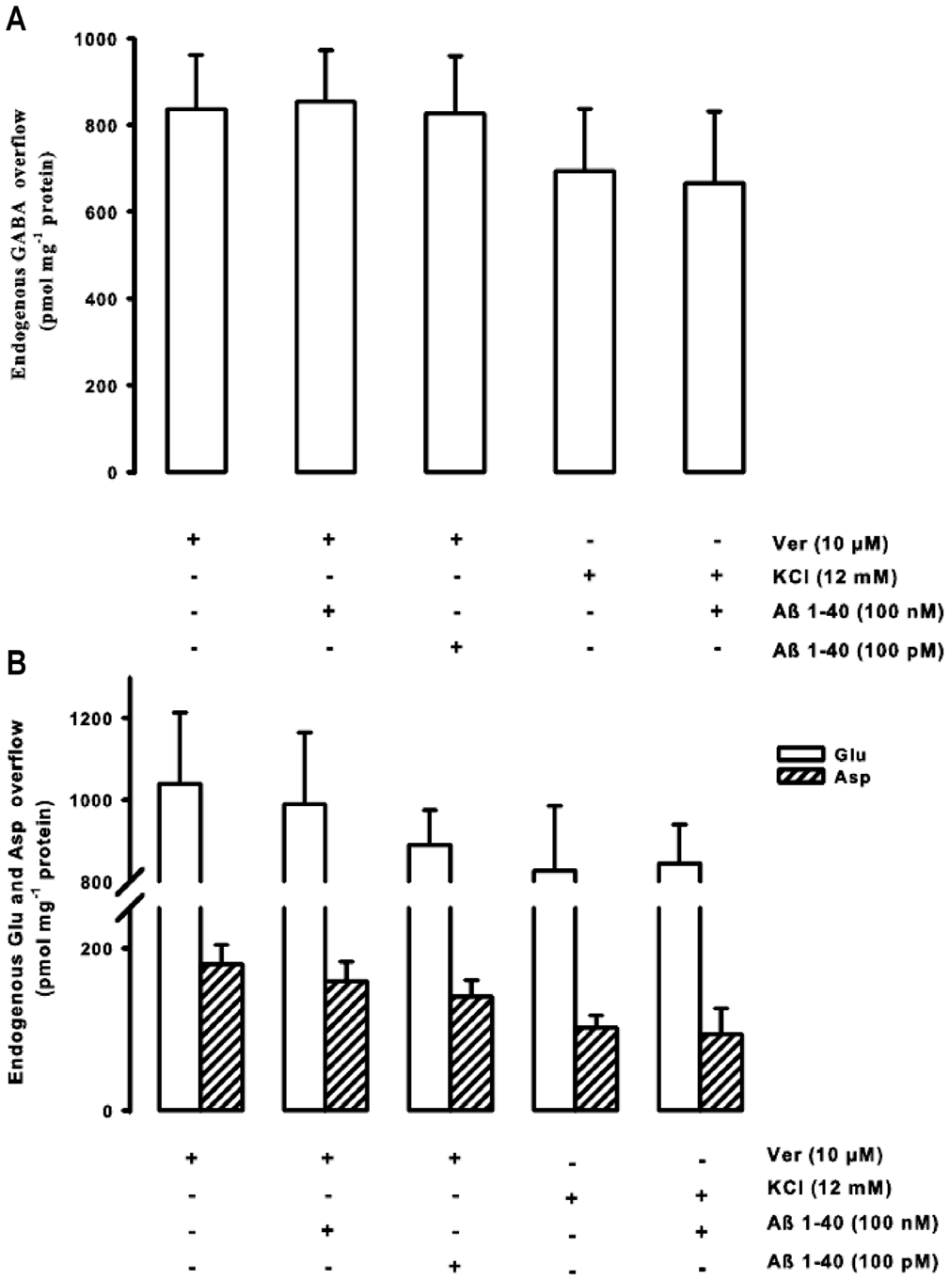
Lack of effect of beta-amyloid on both potassium- and veratridine-induced neurotransmitter release in hippocampus *in vitro*. Effect of different concentrations of beta-amyloid (Aβ)1–40 on both potassium (K^+^)- and veratridine (Ver)-evoked overflow of endogenous GABA (**A**), glutamate and aspartate (Glu and Asp respectively, **B**) from rat hippocampal synaptosomes. Synaptosomes were depolarized either with Ver or with K^+^ for 90 s at *t* = 38 min of superfusion. When appropriate Aβ was introduced 8 min before Ver or K^+^. Data are mean ± SEM of 3–6 experiments run in triplicate.

Parallel to *in vivo* experiments, we also evaluated the effect of Aβ1–40 on the transmitter release stimulated by Ver *in vitro*. 10 µM Ver stimulated an overflow of GABA that was 836.81±124.66 pmol mg^−1^ protein (Fig. 6A). In regard to excitatory neurotransmitters, the Ver-induced overflow of Glu and Asp was 1039.28±174.84 and 179.26±24.49 pmol mg^−1^ protein, respectively (Fig. 6B). Similarly to the *in vivo* results, also *in vitro* Aβ1–40 (tested at 100 pM and 100 nM, thus at the lowest and highest concentration that had been evaluated on synaptosomes) did not modify the release of GABA, Glu, and Asp that was stimulated by a depolarizing stimulus such as Ver (Fig. 6). Moreover, we also evaluated the effects of Aβ1–40 on another depolarizing secretory stimulus such as potassium (K^+^). 12 mM K^+^ evoked an overflow of GABA, Glu and Asp that was 694.00±144.00, 827.00±158.00, 102.00±15.00, respectively. Figure 6 shows that 100 nM Aβ1–40 did not modify the K^+^-induced release of GABA, Glu and Asp.

**Figure 7:**
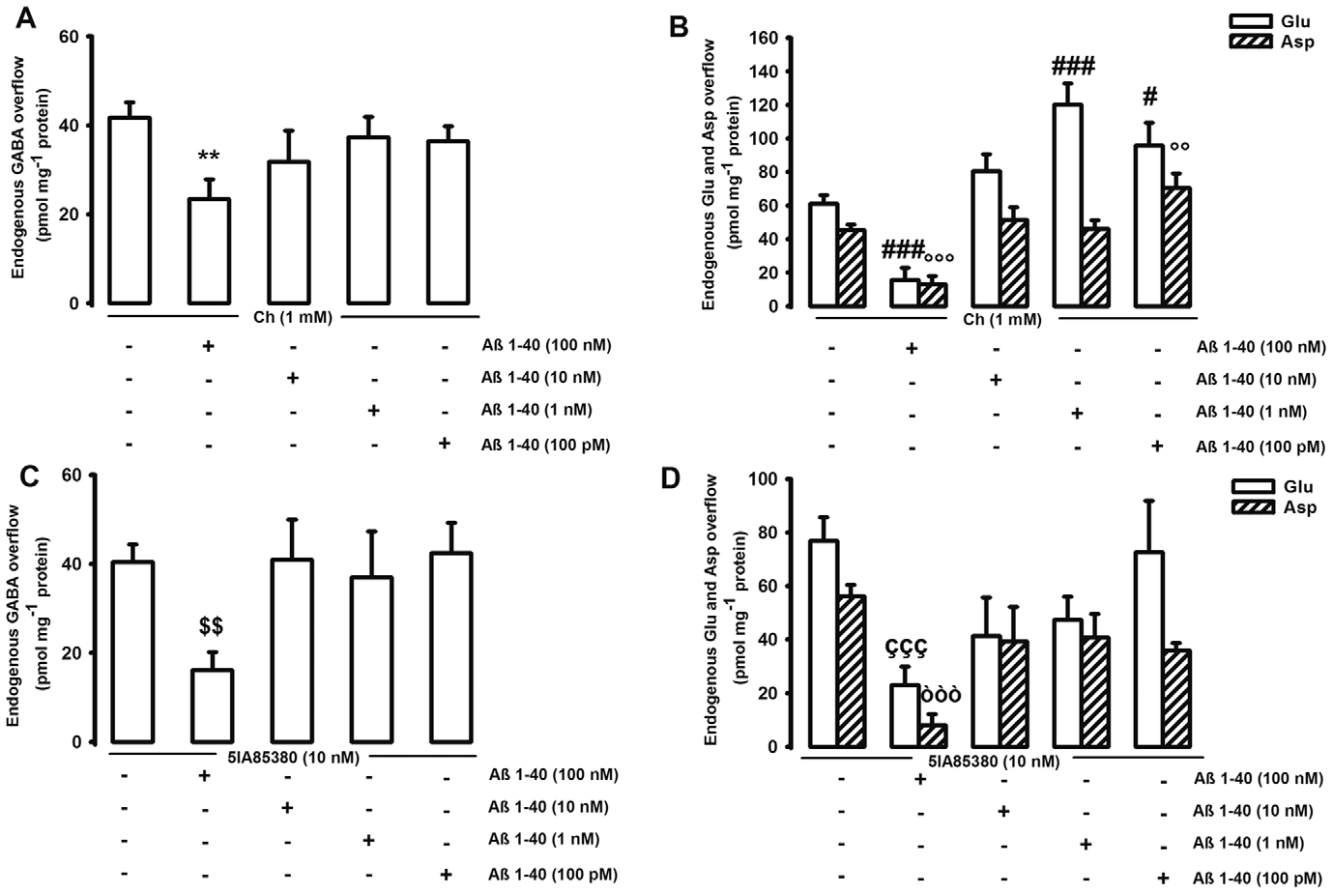
*In vitro* effect of beta-amyloid on hippocampal neurotransmitter release elicited by specific nicotinic agonists. Effect of different concentrations of beta-amyloid (Aβ)1–40 on choline (Ch)-evoked overflow of endogenous GABA (**A**), glutamate and aspartate (Glu and Asp respectively, **B**) and on the 5IA85380-evoked overflow of endogenous GABA (**C**), Glu and Asp (**D**) from rat hippocampal synaptosomes. Synaptosomes were depolarized either with Ch or with 5IA85380 for 90 s at *t* = 38 min of superfusion. When appropriate Aβ was introduced 8 min before the specific nicotinic agonist. Data are mean ± SEM of 3–6 experiments run in triplicate. **p<0.01 vs. Ch-evoked GABA overflow; ^#^p<0.05, ^###^p<0.001 vs. Ch-evoked Glu overflow; ^°°^p<0.01, ^°°°^p<0.001 vs. Ch-evoked Asp overflow. ^$$^p<0.01 vs. 5IA85380-evoked GABA overflow; ^ççç^p<0.001 vs. 5IA85380-evoked Glu overflow; ^òòò^p<0.001 vs. 5IA85380-evoked Asp overflow. One way ANOVA followed by Dunnett *post hoc* test.

Our *in vitro* method (*i.e.,* synaptosomes in superfusion), permits us to unequivocally identify the receptor targeted by specific drugs and anatomically localize it onto a precise synaptosomal population, and to pharmacologically characterize it [30]. Moreover, the possibility that the evoked release of Glu, Asp and GABA may influence the release of other measured neurotransmitters can be almost totally excluded in our experimental set up. Indeed, as previously described, synaptosomes are plated as very thin layers on microporous filters and up-down superfused with physiological solutions (see [30] and references therein). Under these experimental conditions, the transmitters released are removed by the superfusion fluid before they can accumulate and activate presynaptic auto- and heteroreceptors, as well as reuptake carriers, thus excluding the possibility of indirect effects. Using this technique, we recently demonstrated that the hippocampal release of GABA, Glu, and Asp is stimulated by the activation of both α7 and α4β2 nAChRs at presynaptic level [17], [18]. Therefore, using the same experimental conditions, we evaluated whether the effects of Aβ1–40 on the Nic-evoked overflow of GABA, Glu, and Asp may be mediated by either α7 or α4β2 nAChRs or both. In order to do that, we first confirmed the presence and functional effect of both α7 and α4β2 nAChRs on glutamatergic and GABAergic hippocampal nerve endings by using specific agonists. We chose choline (Ch) and 5-Iodo-A-85380 dihydrochloride (5IA85380) as selective agonists for α7 and α4β2, respectively, and we administered them at the same concentration used in our previous studies [17], [18]. In regard to GABA, 1 mM Ch evoked an overflow of 41.69±3.46 pmol mg^−1^ protein that was the same than that elicited by 10 nM 5IA85380 (40.41±3.98 pmol mg^−1^ protein (Figures 7A and 7C). As far as the excitatory neurotransmitters, both 1 mM Ch and 10 nM 5IA85380 evoked an overflow of Glu (61.07±5.22 and 76.91±8.86 pmol mg^−1^ protein respectively) and of Asp (45.52±3.16 and 56.19±4.28 pmol mg^−1^ protein respectively) (Figures 7B and 7D).

There was a dual effect of Aβ1–40 on the release of neurotransmitters evoked by Ch, the selective agonist for α7 nAChR subtype. 100 nM Aβ1–40 (the highest concentration considered *in vitro*) greatly inhibited the Ch-induced overflow of GABA (45%), Glu (75%) and Asp (70%) (Figures 7A and 7B). On the contrary, low concentrations of Aβ1–40 (100 pM and 1 nM) greatly enhanced (100% and 55%, respectively) the Ch-induced Glu release (Fig. 7B). At 100 pM (the lowest concentration considered *in vitro*) Aβ1–40 also potentiated (55%) the Asp release evoked by 1 mM Ch (Fig. 7B). The potentiating effect of low concentrations of Aβ1–40 was not observed in the case of GABA release (Fig. 7A).

As far as the α4β2-selective agonist, 100 nM Aβ1–40 (the highest concentration considered *in vitro*) greatly inhibited the 5IA85380-induced overflow of all three neurotransmitters (GABA = 60%; Glu = 70%; Asp = 85%) (Figures 7C and 7D). All the other concentrations were ineffective.

## Discussion

In the present work we found for the first time that Aβ1–40 affects *in vivo* and *in vitro* the nicotinic-control of the hippocampal release of GABA, Glu, and Asp, in absence of gross signs of neurodegeneration.

Some strategies let us to avoid neurotoxic effects of the peptide. First, the study was focused on Aβ1–40 since it is the most abundant form that is present in physiological state. In fact, the concentration of secreted Aβ1–42 is about 10% that of Aβ1–40 [31]. Moreover, Aβ1–40 and Aβ1–42 have different profiles of aggregation [32]. Aβ1–40 has been reported to aggregate more slowly than Aβ1–42; therefore, the latter is considered as the most neurotoxic species [24]. On the other hand, in our previous *in vivo* experiments in nucleus accumbens, at variance with Aβ1–40, Aβ1–42 was ineffective since it was retained inside the dialysis probe and did not reach the brain tissue, as shown by immunohistochemical analysis [5]. We subsequently administered a freshly prepared solution of Aβ1–40 in order to minimize its aggregation. Finally, we used a dialysis membrane with a cutoff size of 40 KDa to allow the passage through the dialysis fiber of soluble Aβ monomers or small molecular weight oligomers and avoid high molecular weight oligomers (the neurotoxic species as shown by [33]). In our experimental conditions, the peptide did not aggregate (Fig. S1), therefore it did not give origin to neurotoxic oligomeric species.

Following the administration of different concentrations of Aβ1–40 (100 nM–10 µM), immunohistochemical analysis shows that the peptide diffuses through the dialysis membrane to the hippocampal tissue where it is found in proximity of the dialysis probe (Fig. 1). No apoptotic-related phenomena were observed within the area of amyloid diffusion as shown by Hoechst staining. However, we cannot exclude the presence, even at this early time, after Aβ treatment, of more subtle signs of toxicity such as synaptic degeneration and neurite retraction.

### *In vivo* and *in vitro* inhibition of Glu and Asp release by Aβ1–40 and dual effects on GABA release

We observed both *in vivo* and *in vitro* that high concentrations of Aβ1–40 (10 µM and 100 nM, the two highest concentrations respectively used *in vivo* and *in vitro*) greatly inhibit the Nic-induced release of GABA, Glu, and Asp in the hippocampus (Figures 2 and 5). The observed inhibitory effect is consistent with that previously described in the nucleus accumbens and striatum at similar concentrations when studying dopamine and GABA release [5]–[7]. The inhibitory effect on Glu and Asp but not on GABA release was observed also using lower *in vivo* concentrations (1 µM) of administered Aβ (Fig. 2). Surprisingly enough, *in vivo* 100 nM Aβ1–40 (the lowest concentration considered) was able to potentiate the GABA overflow evoked by 50 mM Nic (Fig. 2). To our knowledge, this is the first demonstration of the Aβ1–40 capability to modulate both positively and negatively the nicotinic cholinergic control of GABA release *in vivo*. The observed dual effect of the peptide is in line with the hypothesis that Aβ may have different biological effects increasing the concentrations, possibly in a continuum from physiology to pathology [4]. Interestingly, 100 nM Aβ1–40 potentiates the Nic-induced GABA release (function), 1 µM Aβ1–40 is ineffective (loss of function) and 10 µM Aβ1–40 has an inhibitory effect (gain of new function). The dual effect of Aβ on Nic-induced GABA release was not appreciated *in vitro*.

It is difficult to compare *in vivo* and *in vitro* results for many reasons. First, *in vivo* concentrations are higher than those used *in vitro* in order to guarantee the delivery to the tissue of sufficient amount of drugs. Despite the fact that immunohistochemical micrographs (Fig. 1) show that there is a positive correlation among the Aβ concentrations administered and the amount of immunostaining in the hippocampal tissue, we do not know the exact amount of Aβ that reaches the tissue during a microdialysis experiment and its accumulation/disposal with time. Another difference between the two experimental models is the timing of exposure to experimental drugs (few seconds *in vitro* versus 40 minutes *in vivo*). Moreover, the observation of an *in vivo* potentiating effect of low concentrations of Aβ on the Nic-induced GABA release may be explained by indirect mechanisms of control of neurotransmitter release present *in vivo*. In fact, the levels of both excitatory and inhibitory neurotransmitters measured *in vivo* are the final result of the interactions of hierarchically organized synapses ultimately controlling Glu, Asp, and GABA release. In this regard, the *in vivo* GABA increase mediated by low concentrations of Aβ could be due to an indirect modulatory role of Glu and/or Asp; indeed, we have demonstrated that low Aβ concentrations potentiate the release of Glu and Asp from synaptosomes, which might in turn stimulate GABA release through the activation of glutamatergic receptors on GABAergic neurons [34]. On the contrary, indirect control mechanisms of neurotransmitter release are excluded in our *in vitro* model of synaptosomes in superfusion. In fact, *in vitro* data obtained on perfused synaptosomes are due to the direct effects of the added drugs, which have to act upon receptors or modulatory sites located on the same synaptosome from which occurs the release of the transmitter [30].

### Effect of Aβ on Glu, Asp and GABA release elicited by depolarizing stimuli

*In vivo* and *in vitro* both low and high concentrations of the peptide did not modify the overflow of Glu, GABA, and Asp that was elicited by a depolarizing stimulus such as Ver (Fig. 3 and 6). This observation is consistent with our previous *in vivo* and *in vitro* data demonstrating that neurotransmitter release enhanced by another depolarizing stimulus, such as K^+^, was not affected by Aβ in two different brain areas [5], [6], even if it should be mentioned that Lee and Wang [35] and Kar and colleagues [36] (the latter using hippocampal slices) found an inhibitory action of low Aβ concentrations. Ver is a lipid-soluble neurotoxin that is an activator of Na^+^ channels. It targets the neurotoxin receptor site 2 and preferentially binds to activated Na^+^ channels causing persistent activation [37]. This persistent Na^+^ induces bursts of action potentials and sustains membrane depolarization, which is subsequently correlated with an opening of voltage-gated Ca^2+^ channels [38]. The final result is the enhancement of neurotransmitter release [39]. In our experimental setting, the lack of effect of Aβ on Ver stimulus suggests that Aβ1–40 may affect the Nic-triggered neurotransmitter release directly binding to nAChRs or acting to substrates that are specific for nAChRs-induced intracellular signaling. The action is specific for Aβ1–40 sequence since the reverse peptide was ineffective (Fig. 2 and 5).

### *In vitro* pharmacological dissection of the effect of Aβ on Glu, Asp and GABA release elicited by specific nicotinic agonists

Another possible explanation for the differences of Aβ effect on GABA release observed *in vivo* (dual effect) and *in vitro* (inhibition only) may depend on the differential contribution to the described effect of α7 and α4β2 nAChRs. This aspect was approached *in vitro* by using specific nicotinic agonists. We previously demonstrated that hippocampal glutamatergic and GABAergic nerve endings show both α7 and α4β2 nAChRs that are capable to control neurotransmitter release [17], [18]. The reported results show that the stimulation of both α7 and α4β2 nAChRs elicits the release of Asp, Glu, and GABA, thus indicating that both receptors are positively linked to the release of the studied neurotransmitters, even if it is possible that they reside on different nerve endings populations and that they operate through distinct cellular mechanisms [40]. At high concentrations (100 nM), Aβ is always inhibitory on the release stimulated by both Ch (α7 selective agonist) and 5IA85380 (selective for α4β2 nAChRs) (Fig. 7). On the contrary, low concentrations of Aβ1–40 (1 nM and 100 pM) selectively act on α7 subtypes potentiating the Ch-induced release of both Asp and Glu (Fig. 7B), but not the one elicited by 5IA85380. Therefore, we were able to observe dual effects of Aβ *in vitro* when using specific nAChRs agonists. However, somewhat surprisingly, the stimulatory activity of low Aβ concentrations was associated with Glu and Asp and not with GABA release, as expected. Interestingly, some papers show that the physiological range of concentrations of the peptide is from pM to low nM [41], [42]. Hence, the relevance of our data to AD is that high, likely not physiological, concentrations of Aβ greatly impair cholinergic responses mediated by two different type of nAChRs. On the contrary, physiological concentrations of the peptide may potentiate the positive nicotinic control of neurotransmitter release, specifically acting on α7 subtypes. This last observation is consistent with some data showing the capability of picomolar Aβ to activate α7 receptors currents [43]. This view contrasts with the fact that we did not observe an impact of Aβ on basal neurotransmitter release from synaptosomes. On the other hand, the possibility of a direct activation on α7 receptors by the peptide is consistent with the demonstration that a low picomolar concentration of Aβ enhances hippocampal long term potentiation (LTP) and memory with a mechanism dependent upon activation of α7 nAChRs [44]. Interestingly, Puzzo and collaborators also showed that, physiologically, the presence of the peptide is required for the modulation of LTP and memory formation in a mechanism dependent on α7 nAChRs [45].

Our data show that low concentrations of Aβ (100 pM and 1 nM) seem to selectively modulate α7-depending functions whereas 100 nM Aβ interacts with both receptors subtypes negatively affecting their function. In this regard, Wang and collaborators [8] showed that the peptide can bind with picomolar affinity to α7 subtypes and with 100–5000 times lower affinity to α4β2 nAChRs, suggesting that, in our experimental conditions, a possible mechanism of action of Aβ concerns the physical interaction to nAChRs. Particularly, it seems that Aβ binds nAChRs near the nicotine binding site [46]. However, it has also been hypothesized that Aβ influences the function of α7 subtype by altering the packing of lipids within the plasma membrane, instead of directly binding to the receptors [47]. Since some authors have shown the capability of neurons to internalize Aβ [48], it cannot be excluded that the peptide may enter synaptosomes and act on cytosolic substrates downstream nAChRs.

Our results are at partial variance with those published by Mehta and colleagues [49] showing that in the hippocampus low concentrations of Aβ1–42 increase pre-synaptic Ca^++^ through the action on α4β2 nAChRs, whereas in cortex the involved receptors are mainly the α7 subtypes. The two experimental models, however, are too different to allow a direct comparison. Mehta and colleagues used knockout mice (C57Bl/6J) to demonstrate the selective involvement of nAChRs and measured intrasynaptosomal calcium fluxes, while we measured the release of transmitters altered by the treatment with Aβ1–40 using Wistar rat synaptosomes. As already discussed, in our case we also observed important differences between *in vivo* (in a “wired” system) and *in vitro* experiments. Moreover, it is possible that in knockout mice adaptive phenomena take place with time. In spite of so many differences, it is noteworthy that both our data and those by Mehta and collaborators agree on the possibility that low concentrations of Aβ peptides may stimulate synaptosomal activity through the interaction with nAChRs. Both sets of data show that Aβ peptides may act differently on α4β2 and α7 nAChRs depending on the adopted experimental settings. Interestingly, when using hyppocampal synaptosomes derived from non-transgenic rats the same group of Mehta found that picomolar Aβ directly activates presynaptic α7 nAChRs to increase nerve terminal Ca^2+^ [50] in line with our results.

In regard to the functional effect of the peptide, the dual action of low and high Aβ concentrations on α7-mediated neurotransmitter release may be explained by different ways. First, a desentization-related mechanism, as concentrations of Aβ1–40 in the range 10 pM–1 nM have been reported to activate α7 nAChRs, whereas an higher concentration (100 nM) induces desentization of the receptor [43]. Moreover, increasing the concentrations Aβ may change its targets. In fact, at low concentrations Aβ can selectively activate α7 nAChRs. However, at high concentrations the peptide may act simultaneously on different targets, possibly downstream nAChRs, leading to the oxidative modification of synaptosomal proteins [51] and perhaps to an inhibitory action on neurotransmitter release.

The lack of potentiating effect of low Aβ concentrations on *in vitro* GABA release elicited by Ch further supports the idea that the *in vivo* observed potentiation is due to indirect mechanisms involving a neuronal circuit. On the other hand, it is difficult to explain why low Aβ concentrations potentiate the α7-stimulated release of Glu and Asp, but not the one of GABA. One possibility is that the synaptosomal release machinery of Glu, Asp, and GABA recruited by α7 stimulation differ, as suggested in the case of the sensitivity of glutamatergic and GABAergic release to botulin toxins [52]. Alternatively, it is either possible that variants of α7 receptors [53] may control the release of Glu, Asp, and GABA, or that the differential pacing in the desensitization mechanism of the nAChRs residing on Glu, Asp, and GABA terminals may affect the action of Aβ.

### Conclusions

Altogether, the results reinforce the concepts that Aβ has relevant neuromodulatory effects, which may span from facilitation to inhibition of stimulated release depending upon the concentration. Of particular interest is the observation that, at least *in vitro*, the nicotinic cholinergic control of two excitatory neurotransmitters, Asp and Glu, was particularly sensitive to the effect of low Aβ concentrations in synaptosomes derived from the hippocampus. Moreover, the involved receptor seems to be the α7 one, which other lines of evidence suggest to be a target of Aβ. These actions may be relevant to the early stages of AD which, rather than being characterized by neurodegeneration, may be associated with synaptic dysfunctions affecting more than a transmitter system, albeit with peculiar sensitivity of mechanisms associated with nicotinic cholinergic transmission. It may be hypothesized that the early derangement of Aβ production may lead to trespass the threshold beyond which Aβ loses the ability to co-promote Asp and Glu release, which may be linked to an efficient memory trace formation, and subsequently gains the ability to directly inhibit the ability of cholinergic stimuli to promote Glu and Asp release. This may further impair the signal to noise ratio of Glu transmission associated with synaptic trace formation. The neurotoxicity may finally eventually become the leading event. Moreover, parallel to this impairment of cholinergic control of Glu and Asp release, other effects may be present in other brain areas involving other neurotransmitters, thus providing the basis for a multitransmitter deficit in the disease. This is turn may be responsible for the various psychiatric symptoms that characterize subsets of patients, in addition to the more easily recognized memory deficits. These observations may help to redirect the pharmacological approaches toward multiple neurotransmitter targets in the early stages of the disease.

## Materials and Methods

### Chemicals

5-Iodo-A-85380 dihydrochloride (3-[(2S)-2-Azetidinylmethoxy]-5-iodopyridine dihydrochloride) was purchased from Tocris Bioscience (Bristol, UK); nicotine hydrogen tartrate salt, veratridine, Aβ1–40 and Aβ40–1, Percoll®, Choline Iodide, dimethyl sulfoxide were obtained from Sigma Aldrich (Milan, Italy); all salts used for the preparation of artificial cerebrospinal fluid (aCSF) (NaCl, KCl, CaCl_2_, MgCl_2_, Na_2_HPO_4_) and for Equithesin (MgSO_4_) were purchased at Merck KGaA, Darmstadt, Germany; chloral hydrate, ethanol 96% and propylene glycol were used for the preparation of Equithesin and were obtained at VWR BDH Prolabo, Belgium.

### Preparation of Aβ1–40 solutions

In the case of both *in vivo* and *in vitro* experiments, synthetic human Aβ1–40 (Sigma Aldrich, Milan, Italy) was dissolved in aCSF at a concentration of 100 µM (stock solution). Then, this solution was filtered through a Millipore 0.2 µm pore membrane and stocked in small aliquots at −80°C. Working solutions were freshly prepared by diluting an aliquot of Aβ1–40 stock solution at the final concentrations (10 µM, 1 µM, or 100 nM Aβ1–40 for *in vivo* experiments, 100 nM, 10 nM, 1 nM, or 100 pM for *in vitro* analysis).

### Animals

Young male Wistar rats (275–300 g; Harlan, Udine Italy), housed in standard conditions (temperature 23±1°C; humidity 50%) with 12∶12 light/dark cycles, water and food *ad libitum*, were used either for microdialysis experiments or as brain tissue source for the *in vitro* experiments. The use of animals for the preparation of synaptosomes was approved by the Ethical Committee of the Pharmacology and Toxicology Section, Department of Experimental Medicine, in accordance with the European legislation (European Communities Council Directive of 24 November 1986, 86/609/EEC) and were approved by Italian legislation on animal experimentation (Decreto Ministeriale number 124/2003-A). The *in vivo* protocol was approved by Ethical Committee of Pavia's University (registered as 2/2008) according to international regulations for the care and treatment of laboratory animals, to the Italian Act (DL n 116, GU, suppl 40, 18 February, 1992) and to EEC Council Directive (86/609, OJ L 358, 1, 12 December, 1987). All efforts were made to minimize animal suffering and to use the minimal number of animals necessary to produce reliable results.

### *In vivo* experiments

#### Microdialysis probe implantation

Rats were anesthetized with Equithesin 3 ml/kg (pentobarbital 9.7 g, chloral hydrate 42.5 g, MgSO_4_ 21.3 g for 1 L, 10% ethanol, 40% propylene glycole v/v) administered intraperitoneally and placed in a stereotaxic apparatus (David Kopf Instruments, Tujunga, CA, USA). The skin was shaved, disinfected, and cut with a sterile scalpel to expose the skull. A hole was drilled to allow the implantation of the probe into the brain parenchyma. The probe was implanted in the hippocampus (CA1/CA2 regions; AP −5.8 mm, ML ±5.0 mm from bregma and DV–8.0 mm from dura, according to [54]) and secured to the skull with one stainless steel screw and dental cement. All experiments were performed using microdialysis probes, made in our laboratory according to the original method described by Di Chiara and colleagues [55] (Emophan Bellco Artificial OR-internal diameter 200 µm, cutoff 40 KDa; Bellco, Mirandola, Italy), with a nominal active length of 5 mm.

Finally, the skin was sutured, and the rats were allowed to recover from anesthesia.

#### Microdialysis samples collection

Microdialysis experiments were performed on conscious freely moving rats. On the day of the experiments (24 hours after the surgical procedure), the probe was perfused with aCSF containing 145 mM NaCl, 3.0 mM KCl, 1.26 mM CaCl_2_, 1.0 mM MgCl_2_, 1.4 mM Na_2_HPO_4_, buffered at pH 7.2–7.4 and filtered through a Millipore 0.2 µm pore membrane. In all experiments, the microdialysis membrane was allowed to stabilize for 1 hour at the flow rate of 4 µl/min, without collecting samples. At the end of the stabilization period, three samples were collected to evaluate baseline release of GABA, Asp, and Glu; then, the specific treatment started. In the case of Aβ administration, a pre-treatment of 20 min with the peptide preceded its co-perfusion either with Nic or with Ver. Anyway, the perfusion of the peptide did not last more than 100 min. All treatments were administered by manually switching syringes and tubing connections to allow drugs diluted in aCSF to flow through the probes. Tubing switches were performed taking care to maintain constant flow rates and collection volumes. Both basal and treatment samples were collected every 20 min in 100 µl Eppendorf tubes at a flow rate of 4 µl/min, using a 1000 µl syringe (Hamilton) and a microinjection pump (CMA/100, CMA/Microdialysis AB). Levels of GABA, Glu and Asp in the dialysate were measured by high performance liquid chromatography (HPLC) with fluorometric detection (see **Chromatography**). *In vitro* recovery of the dialysis probe was 13.88±0.25 for Asp, 15.21±0.42 for Glu and 7.91±0.25 for GABA. Each rat was used for only one microdialysis session. At the end of each experiment, animals were sacrificed by guillotine, rat brains were removed, and the position of the microdialysis probe was verified by histological procedures, slicing the tissues by a cryostat microtome (LEICA CM 1510). Only data from rats in which probe tracks were exactly located in the target area were used for statistical analysis.

### *In vitro* experiments

#### Experiments of release

Rats were killed by decapitation and the hippocampus was rapidly removed at 0–4°C. Purified synaptosomes were prepared on Percoll® gradients (Sigma-Aldrich, St Louis, MO, USA) according to the original method described by Dunkley et al., [56], with only minor modifications. Briefly, the tissue was homogenized in 6 volumes of 0.32 M sucrose, buffered at pH 7.4 with Tris–HCl, using a glass-teflon tissue grinder (clearance 0.25 mm, 12 up–down strokes in about 1 min). The homogenate was centrifuged (5 min, 1000 g at 4°C) to remove nuclei and debris; the supernatant was gently stratified on a discontinuous Percoll® gradient (2%, 6%, 10%, and 20% v/v in Tris-buffered sucrose) and centrifuged at 33500 g for 5 min at 4°C. The layer between 10% and 20% Percoll® (synaptosomal fraction) was collected, washed by centrifugation and resuspended in physiological HEPES-buffered medium having the following composition (mM): NaCl 128, KCl 2.4, CaCl2 3.2, KH2PO4 1.2, MgSO4 1.2, HEPES 25, pH 7.5, glucose 10, pH 7.2–7.4 [57]. Synaptosomal protein content following purification was 10–15% of that in the supernatant stratified on the Percoll® gradient.

The synaptosomal suspension was layered on microporous filters at the bottom of a set of parallel superfusion chambers maintained at 37°C [30] (Superfusion System, Ugo Basile, Comerio, Varese, Italy). Synaptosomes were superfused at 1 ml/min with standard physiological medium as previously described. The system was first equilibrated during 36.5 min of superfusion; subsequently, four consecutive 90 s fractions of superfusate were collected. Synaptosomes were exposed to agonists for 90 s starting from the second fraction collected (t = 38 min), with antagonists being added 8 min before agonists. We have previously amply demonstrated that in our superfusion system the possible effects of drugs operated indirectly by other mediators in the monolayer of synaptosomes in superfusion are absolutely minimized [30].

### Chromatography

In both dialysates and fractions collected from synaptosomes in superfusion levels of endogenous GABA, Glu, and Asp were measured by HPLC analysis following precolumn derivatization with o-phthalaldehyde and resolution through a C18-reverse phase chromatographic column (10×4.6 mm, 3 µm; Chrompack, Middleburg, The Netherlands) coupled with fluorometric detection (excitation wavelength 350 nm; emission wavelength 450 nm). Homoserine was used as internal standard. Buffers and gradient program were prepared and executed as follows: solvent A, 0.1 M sodium acetate (pH 5.8)/methanol, 80∶20; solvent B, 0.1 M sodium acetate (pH 5.8)/methanol, 20∶80; solvent C, sodium acetate (pH 6.0)/methanol, 80∶20; gradient program, 100% C for 4 min from the initiation of the program; 90% A and 10% B in 1 min; 42% A and 58% B in 14 min; 100% B in 1 min; isocratic flow 2 min; 100% C in 3 min; flow rate 0.9 ml/min.

### Immunohistochemical analysis

Immunohistochemical analysis was performed to verify the presence of Aβ in the perfused tissue and to confirm (according to HOECHST 33342 staining) the absence of neurotoxic-induced apoptotic phenomenon. Brain tissue samples were frozen and stored at −80°C. For immunodetection of infused Aβ peptide, 10 µm coronal sections (obtained on a cryostat Leica CM 1510) were incubated with a primary monoclonal antibody recognizing Aβ protein (clone 4G8; Chemicon International). Sections were then incubated with a mouse anti-IgG antibody RPE conjugated (Dako). After the fluorescent labeling procedures, sections were finally counterstained for DNA with HOECHST 33342 and mounted in a drop of Mowiol (Calbiochem, Inalco SpA, Milan, Italy). Fluorescent micrographs were acquired with a Leica TCS SP5 II confocal microscope. After acquisition of fluorescent micrographs, the slides were demounted and then the same sections were slightly counterstained with Mayer hematoxylin, dehydrated and mounted in DPX for microanatomical analysis. The images were acquired with a BX51 Olympus microscope.

### Western blotting procedure

Samples were subjected to SDS-PAGE (15%) and then transferred onto PVDF membrane (DuPont NEN, Boston MA). The membrane was blocked for 1 hour with 5% non fat dry milk in Tris-buffered saline containing 1% Tween 20 (TBST). Membranes were immunoblotted with the antibody 6E10, recognizing residues 1–17 of Aβ (Chemicon-Prodotti Gianni, Milano, Italy). The detection was carried out by incubation with horseradish peroxidase conjugated goat anti-mouse IgG (Kirkegaard and Perry Laboratories, Gaithersburgh, MD U.S.A.) for 1 h. The blots were then washed extensively and Aβ visualized using an enhanced chemiluminescent methods (Pierce, Rockford, IL, USA). Molecular mass was estimated by molecular weight markers (Invitrogen).

### Statistical analysis

#### *In vivo* experiments

Values were expressed either as amount of neurotransmitter measured in the dialysate (pmol/80 µL) or as area under the curve (AUC), evaluating the cumulative release over time. AUC was used as a measure of treatment exposure and was calculated for each animal using GraphPad Prism (version 4.03 GraphPad Software, San Diego, CA, USA). The basal value (average concentration of three consecutive samples immediately preceding the drug dose) was used as baseline to calibrate the calculation.

D'Agostino-Pearson Omnibus Test (GraphPad Prism, version 4.03, GraphPad Software, San Diego, CA, USA) and Grubb's Test (GraphPad QuickCalcs, online calculator for scientists at http://www.graphpad.com/quickcalcs/, GraphPad Software, San Diego, CA, USA) were used as preliminary tests in order to evaluate whether data were sampled from a Gaussian distribution and to detect outliers respectively. All outliers were excluded from the analysis. Data were analyzed by analysis of variance (ANOVA) followed, when significant, by an appropriate *post hoc* comparison test. Data were considered significant for p<0.05. The reported data are expressed as means ± S.E.M. The number of animals used for each experiment is reported in the legend to Figures 2 and 3.

#### *In vitro* experiments

The evoked overflow was calculated by subtracting the corresponding basal release from each fraction. All data are expressed as pmol·mg^−1^ protein and represent mean ± SEM of the number of experiments reported in the figure legends. Multiple comparisons were performed with one-or two way ANOVA followed by an appropriate post hoc test (Dunnett or Bonferroni). Data were considered significant for p<0.05 at least, using KyPlot 2.0 beta 15.

## Supporting Information

Figure S1 **Characterization of beta-amyloid (Aβ) conformation by using Western Blot procedure.** SDS-PAGE showing immunoreactive species consistent with Aβ monomer in all the preparations analyzed: the stock solution (100 µM Aβ1–40) freshly prepared, the stock solution (100 µM Aβ1–40) maintained for 100 min at room temperature and the most concentrated working solution evaluated *in vivo* (10 µM Aβ1–40) maintained for 100 min (maximum length of Aβ1–40 perfusion during microdialysis experiments) at room temperature.
(TIF)

Figure S2 **Lack of effect of beta-amyloid on the basal neurotransmitter release in hippocampus** ***in vivo*** **.** Effect of beta-amyloid (Aβ)1–40 (100 nM–10 µM) on the basal release of GABA (**A**), glutamate (Glu, **B**) and aspartate (Asp, **C**). One-way ANOVA. Data are expressed as mean ± SEM of 4–9 individual rats for each experimental group.
(TIF)
