# Supplementary figures and images for: Dual Effect of Beta-Amyloid on α7 and α4β2 Nicotinic Receptors Controlling the Release of Glutamate, Aspartate and GABA in Rat Hippocampus
Source: PLoS One. 2012 Jan 11;7(1):e29661. doi: 10.1371/journal.pone.0029661 (PMC3256170; doi:10.1371/journal.pone.0029661)

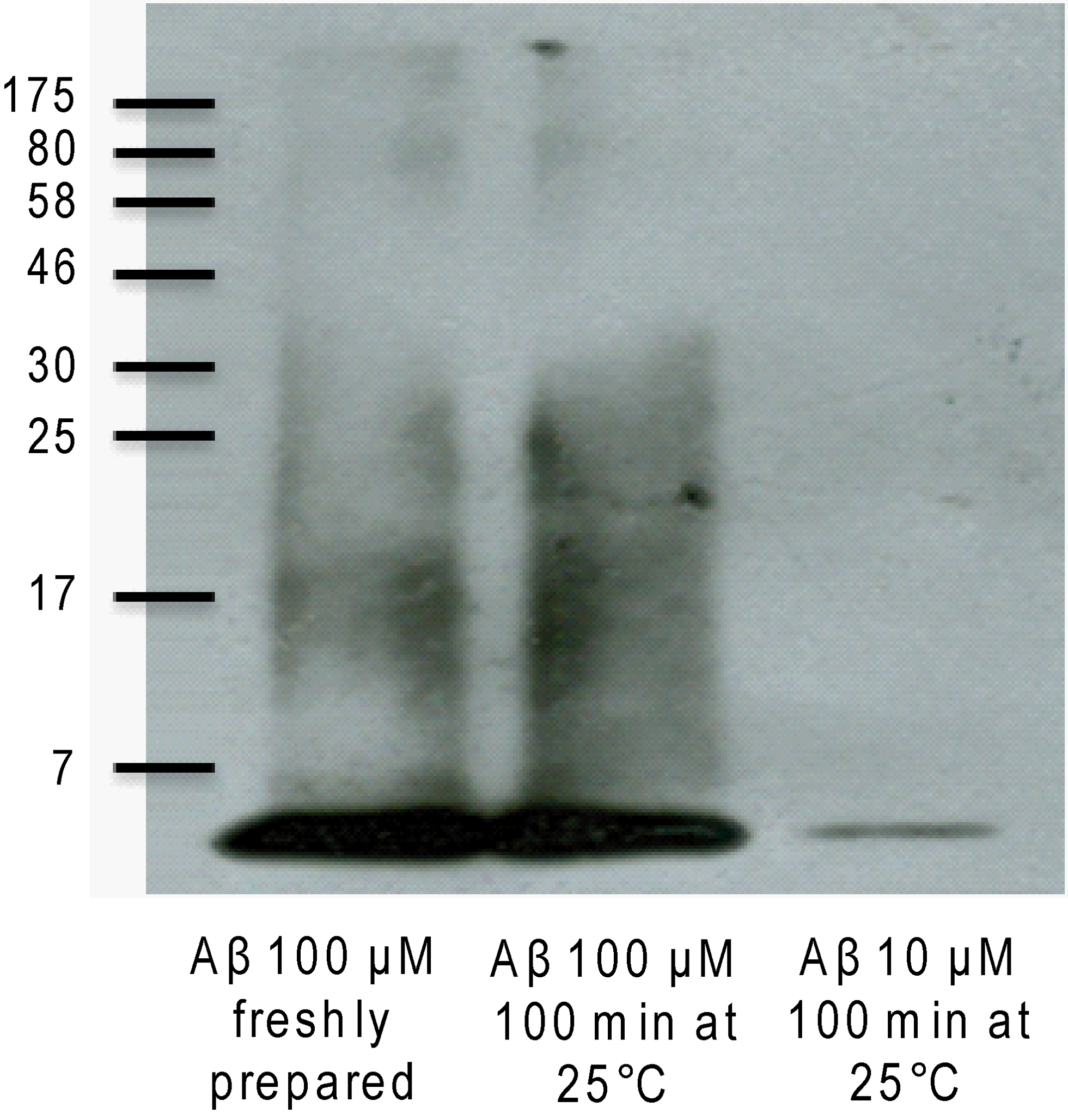

Supplement: Figure S1 — Characterization of beta-amyloid (Aβ) conformation by using Western Blot procedure. SDS-PAGE showing immunoreactive species consistent with Aβ monomer in all the preparations analyzed: the stock solution (100 µM Aβ1–40) freshly prepared, the stock solution (100 µM Aβ1–40) maintained for 100 min at room temperature and the most concentrated working solution evaluated in vivo (10 µM Aβ1–40) maintained for 100 min (maximum length of Aβ1–40 perfusion during microdialysis experiments) at room temperature. (TIF) [file pone.0029661.s001.tif]

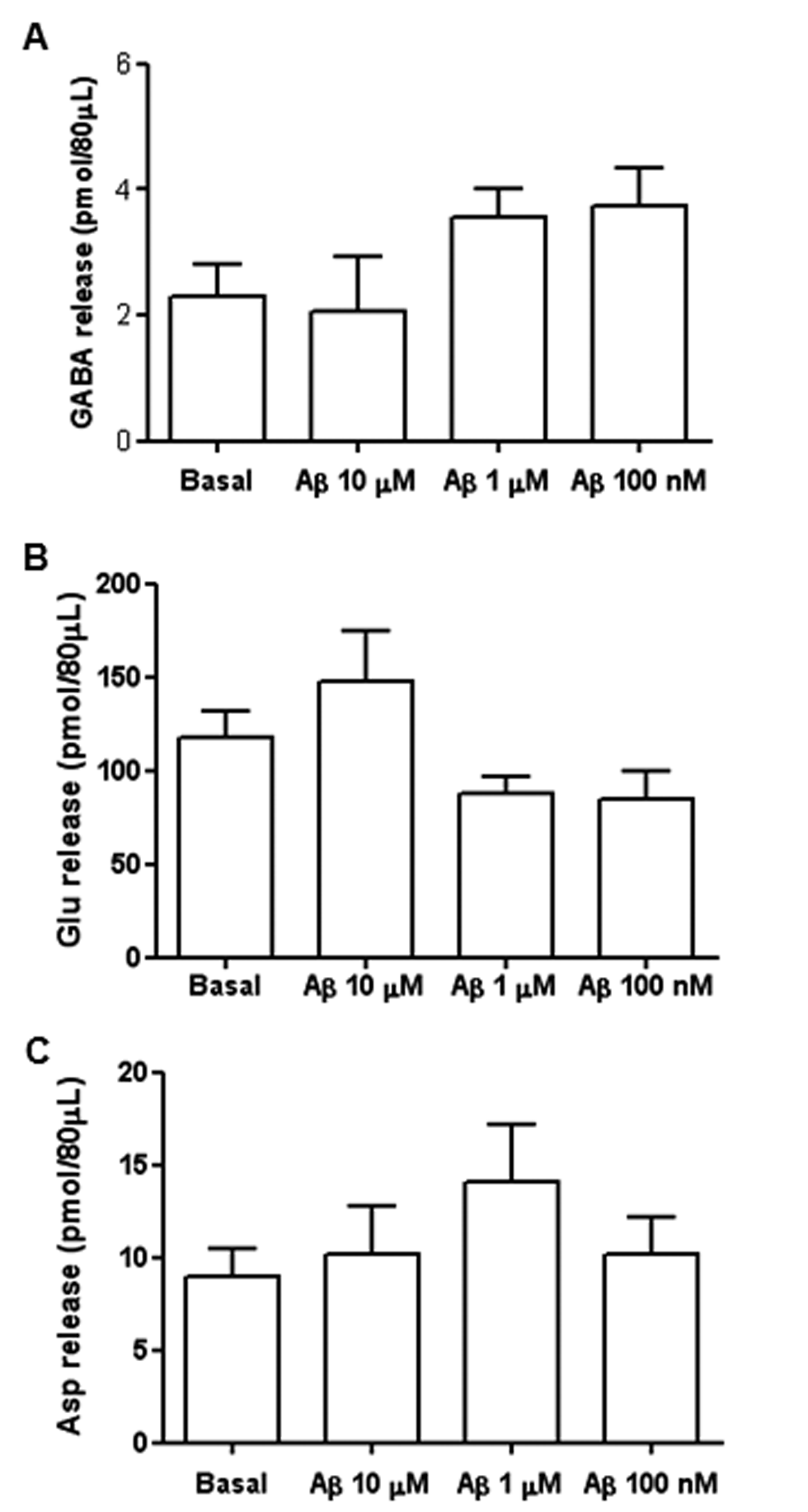

Supplement: Figure S2 — Lack of effect of beta-amyloid on the basal neurotransmitter release in hippocampus in vivo . Effect of beta-amyloid (Aβ)1–40 (100 nM–10 µM) on the basal release of GABA (A), glutamate (Glu, B) and aspartate (Asp, C). One-way ANOVA. Data are expressed as mean ± SEM of 4–9 individual rats for each experimental group. (TIF) [file pone.0029661.s002.tif]
